# Scaffold-free approach produces neocartilage tissue of similar quality as the use of HyStem™ and Hydromatrix™ scaffolds

**DOI:** 10.1007/s10856-017-5870-2

**Published:** 2017-02-16

**Authors:** Janne H. Ylärinne, Chengjuan Qu, Mikko J. Lammi

**Affiliations:** 1 Department of Integrative Medical Biology, Umeå University, Umeå, Sweden; 2 School of Public Health, Health Science Center of Xi’an Jiaotong University, Key Laboratory of Trace Elements and Endemic Diseases, National Health and Family Planning Commission, Xi’an, P. R. China

## Abstract

**Abstract:**

Numerous biomaterials are being considered for cartilage tissue engineering, while scaffold-free systems have also been introduced. Thus, it is important to know do the scaffolds improve the formation of manufactured neocartilages. This study compares scaffold-free cultures to two scaffold-containing ones. Six million bovine primary chondrocytes were embedded in HyStem™ or HydroMatrix™ scaffolds, or suspended in scaffold-free chondrocyte culture medium, and then loaded into agarose gel supported culture well pockets. Neocartilages were grown in the presence of hypertonic high glucose DMEM medium for up to 6 weeks. By the end of culture periods, the formed tissues were analyzed by histological staining for proteoglycans (PGs) and type II collagen, gene expression measurements of aggrecan, Sox9, procollagen α_1_(II), and procollagen α_2_(I) were performed using quantitative RT-PCR, and analyses of PG contents and structure were conducted by spectrophotometric and agarose gel electrophoretic methods. Histological stainings showed that the PGs and type II collagen were abundantly present in both the scaffold-free and the scaffold-containing tissues. The PG content gradually increased following the culture period. However, the mRNA expression levels of the cartilage-specific genes of aggrecan, procollagen α_1_(II) and Sox9 gradually decreased following culture period, while procollagen α_2_(I) levels increased. After 6-week-cultivations, the PG concentrations in neocartilage tissues manufactured with HyStem™ or HydroMatrix™ scaffolds, and in scaffold-free agarose gel-supported cell cultures, were similar to native cartilage. No obvious benefits could be seen on the extracellular matrix assembly in HyStem™ or HydroMatrix™ scaffolds cultures.

**Graphical abstract:**

## Introduction

Today, tissue engineering is considered to offer great possibilities for the field of regenerative medicine, and researchers working on various disciplines of the regenerative medicine show great interest on it worldwide. The principle of the tissue engineering is to regenerate neotissues by using cells, biomaterials and/or signaling molecules to replace the lost or damaged tissue. Although there has been some limited success, lately some promising new results have been introduced [1, 2].

Articular cartilage is basically quite simple tissue in its structure and function. Therefore, it seems to be one of the most ideal candidates for the tissue engineering approaches [3]. Yet, there are no standardized methods available at present to reproducibly succeed in the regeneration of a perfect functional articular cartilage in vitro*.* Obviously, the problem lies in the ability to create a correct fine structure of the tissue extracellular matrix. It is relatively easy to grow a cartilage-like tissue in laboratory conditions, but usually the tissues lacks a zonal organization and resilience of the true articular cartilage [4, 5].

The development of better culture methods is often achieved by testing new protocols step by step. By systematically going through the different options, a true knowledge of the steps of importance can be gathered. Earlier, we have optimized our neocartilage culture conditions using a scaffold-free culture system. Contrary to our hypothesis, we noticed that low oxygen tension and glucosamine sulphate supplementation were not beneficial for the neocartilage formation in a three-dimensional scaffold-free culture system [6], although 5% oxygen in monolayer cultures was advantageous for gene expressions of aggrecan and procollagen α_1_(II) [7]. On the other hand, a hypertonic (390 mOsm) high glucose (4.5 g/l) culture medium under normal oxygen tension (20% O_2_) enhanced the neocartilage formation in the scaffold-free primary chondrocyte culture system [8]. Analyses based on histological stainings, relative cartilage-specific gene expressions and proteoglycan (PG) contents showed that these culture conditions were better than the regular culture conditions [8]. Notably, a transient transforming growth factor-β_3_ (TGF-β_3_) supplementation revealed no benefit at all when compared to the non-supplemented cultures under the same conditions [8], even though it has been shown previously that the transient supplementation of TGF-β_1_ or TGF-β_3_ stimulated the extracellular matrix synthesis of the tissue-engineered cartilage [9].

In our previous studies, irregular shapes of the neocartilages were often formed, and especially the surface profiles could be variable. In this study, our goal was to optimize our culture method by embedding the chondrocytes into commercial scaffold materials HyStem™ and HydroMatrix™. The scaffold materials were expected to aid in the tissue formation, giving the neocartilages support especially at the beginning of the cultivation and, therefore, speed up the process of tissue assembly. The agarose well culture system, similar to those introduced previously [10–12], were considered to provide the neocartilages with better conditions for nutrients and O_2_ supplies and, therefore, prevent the possible necrotic events in the neotissues due to suboptimal culture conditions.

The HyStem™, a synthetic hyaluronan product, is a native material for articular cartilage modified with functional thiol-groups. This allows the scaffold material to be crosslinked using certain crosslinking agent. HydroMatrix™ is a synthetic self-assembling peptide, which can be used to form hydrogels by changes in temperature or in ionic strength. The feasibility of these materials for cartilage tissue engineering was tested in this study in comparison to scaffold-free cultivations.

## Materials and methods

### Preparation of agarose gel wells

**Fig. 1:**
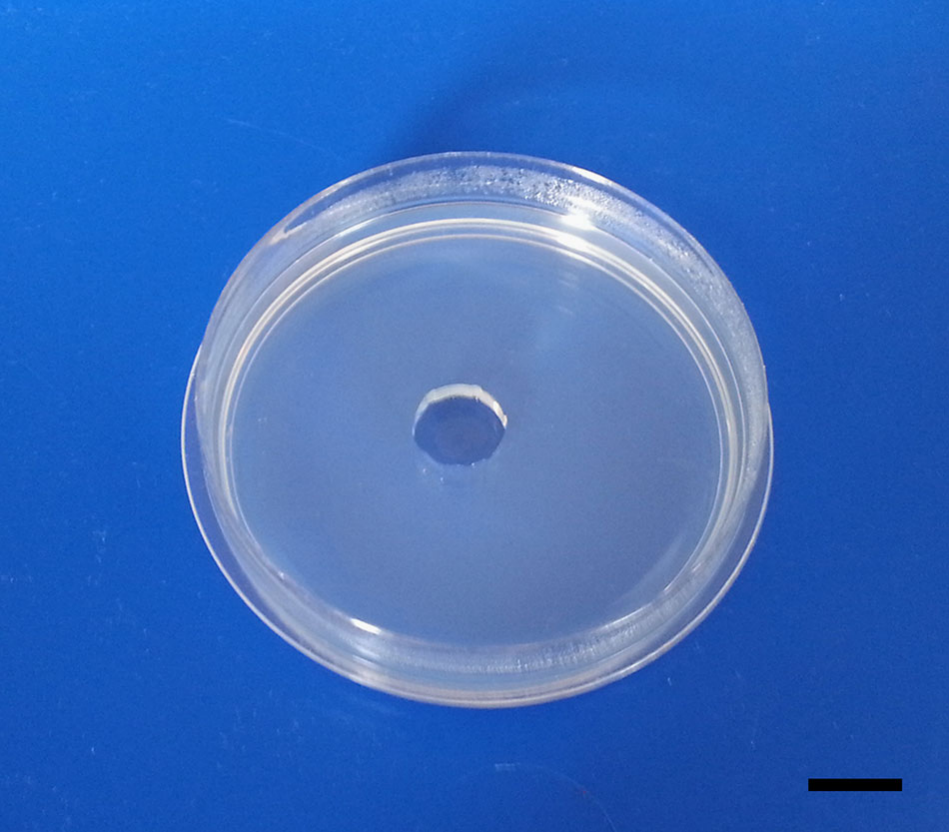
Photograph of agarose gel-support well used in the cultures (scale bar = 1 cm)

First, 1% agarose gel was prepared in phosphate-buffered saline (PBS) and heated in microwave oven. The soluble gel was poured into a 6-cm cell culture plate after a specific cylinder-shaped mold (diameter = 10 mm) was put on the plate to form the wells in the gel. After gelation at room temperature for about 1 h, the mold was taken away, and the gel well plates were ready for cell culture (Fig. 1).

### Isolation of chondrocytes and cell seeding

Cartilage was harvested from bovine femoral condyles provided by HK Scan (Outokumpu, Finland) and Strömdahla (Nordmaling, Sweden) abattoirs. The chondrocytes were isolated by overnight collagenase digestion in high glucose DMEM supplemented with fetal bovine serum, penicillin, streptomycin, L-glutamine and L-ascorbic acid 2-phosphate trisodium salt, as previously described [8].

The next day, six million chondrocytes were seeded into the agarose gel wells in HyStem™ or HydroMatrix™ scaffolds, or as a control without the scaffold. Chondrocytes seeded in the HyStem™ scaffolds were prepared by mixing six million chondrocytes into 100 µl of the HyStem™ solution and adding 25 µl of the crosslinking solution of the kit to start the gelation process. The suspension was immediately gently pipetted into the gel well, and placed into a humidified incubator for 20 min. After the gelation, 5 ml of high glucose hypertonic (390 mOsm) DMEM supplemented with 10% fetal bovine serum, 100 U/ml penicillin, 100 µg/ml streptomycin, 2 mM L-glutamine and 50 µg/ml L-ascorbic acid 2-phosphate trisodium salt was added into plate.

The HydroMatrix™ solution was kept on ice before initiating the gelation, then six million chondrocytes were mixed into the product (100 µl, final concentration 0.25%) and 5 ml of the hypertonic high glucose medium, supplemented as described above, was gently poured into the plate. The sample was then placed into the humidified incubator to start the gelation process and cultivation.

The scaffold-free controls, prepared by suspending six million chondrocytes in 100 µl of the above-described high glucose hypertonic DMEM into the agarose wells, were placed into incubator for 1 h, and then 5 ml of the high glucose hypertonic DMEM was very carefully added to the plate. The samples were grown for 1, 3 and 6 weeks. After each time point, the samples were collected for histological staining, PG analyses and gene expression analyses. Each tissue was also photographed for macroscopical evaluation. The experiments were repeated for four times performing four different chondrocyte isolations from four different animals. For gene expression sample collection, the experiments were repeated for six times.

### Histological assays

At the end of each culture period, one sample from each treatment and control was cut into two halves: one for the PG analyses, while the other half was fixed in 4% paraformaldehyde and processed for the histological analyses. The samples reserved for the PG analyses were weighed in a pre-weighed tubes to obtain the wet weight of the sample, and then frozen in −70 °C for the further analyses.

The histological sections of the samples were stained with Toluidine Blue, and type II collagen was immunostained with anti-type II collagen mouse monoclonal antibody E8 [6, 13]. Envision+ System-HRP kit (Dako, Glostrup, Denmark) was used for detection. The stained sections were then imaged using a light microscope (Carl Zeiss Axioimager M2, Thornwood, NY, USA). The thicknesses of the neotissues were measured from the same sections using the same device using a standard scale bar for calibration.

### Analysis of the proteoglycans

The PGs were extracted with 4 M guanidinium hydrochloride as described in our previous studies [6, 8]. The extracted PGs were precipitated in 70% ethanol, dissolved in water, and glycosaminoglycan (GAG) contents were quantitated using 1,9-dimethylmethylene blue (Sigma-Aldrich) assay [14]. Chondroitin sulfate from shark (Sigma) was used as a standard. Separation of the extracted PGs in a 1.2% agarose gel and staining with Toluidine Blue was performed as previously described [6].

### Gene expression analyses

The gene expression analyses were performed as described in our previous study [8]. Briefly, TRI reagent (Molecular Research Center, Cincinnati, OH, USA) was used to extract the total RNA from each sample, and reverse transcription was performed according to the kit’s instructions (Verso cDNA Synthesis Kit, Thermo Scientific, Waltham, MA, USA). The quantitative RT-PCR was accomplished by using MX3000P Real Time PCR System (Stratagene, La Jolla, CA, USA) as previously described [8] using the primers optimized for aggrecan [15], procollagen α_2_(I) [16], procollagen α_1_(II) [16] and Sox9 [17]. Ribosomal protein large P0 (RPLP0) was used as a house-keeping gene [18].

### Statistical analysis

One-way analysis of variance with the Bonferroni correction was used to analyze the statistically significant differences in the determined parameters between the different time points, and scaffold vs. scaffold-free cultures. A difference was interpreted to be statistically significant when the *P*-value was less than 0.05.

## Results

### Macroscopical appearance and the thickness of the neocartilages

**Fig. 2:**
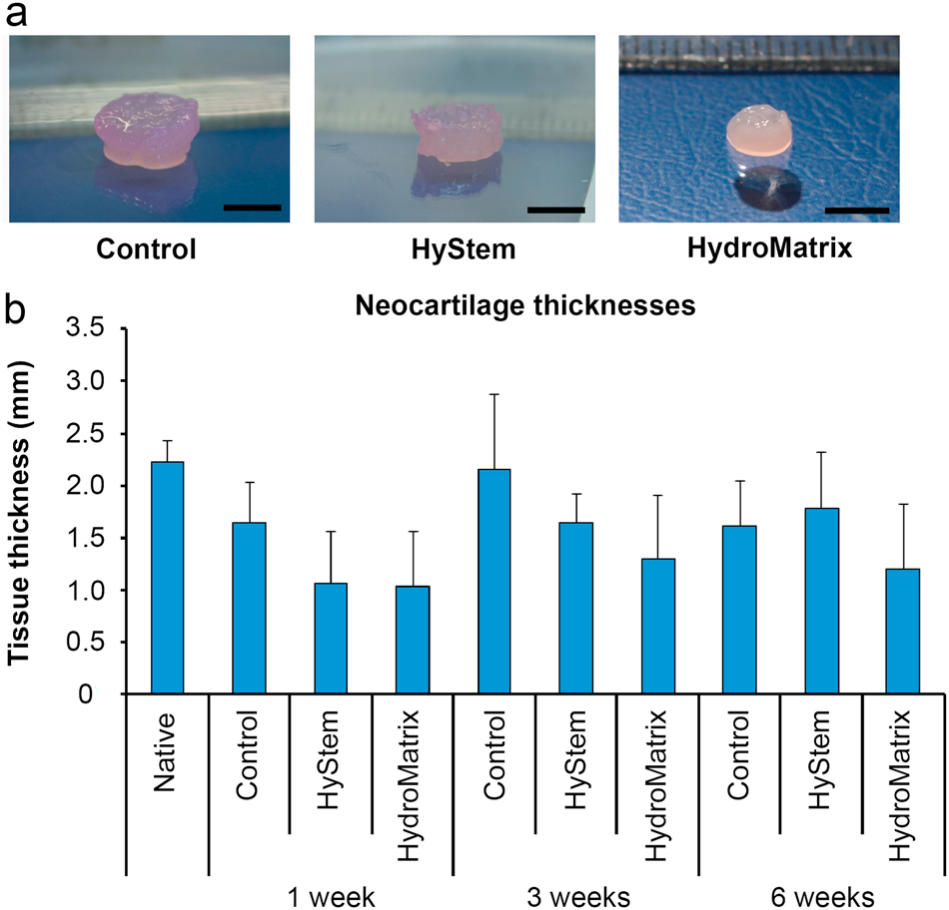
Macroscopical appearance of the neocartilage tissues generated in the HyStem™ or the HydroMatrix™ scaffolds, or the scaffold-free (control) cultures after 6-week culture period (scale bar = 5 mm) **a** The thicknesses (mean ± 95% confidence intervals, *n* = 4) of the neocartilage tissues generated in the HyStem™ or the HydroMatrix™ scaffolds or the scaffold-free (Ctrl) cultures after 1, 3 or 6 weeks of culture **b** *Native:* thickness of native bovine articular cartilage tissue (mean ± 95% confidence intervals, *n* = 3)

Overall, the control and the HyStem™ samples were disc-shaped and smoothest on the surface, whereas the tissues grown in the HydroMatrix™ scaffolds were more often irregularly shaped, and their surfaces were not as smooth as in the control ones. Furthermore, the tissues grown in the control cultures appeared slightly larger in size than the ones grown with the scaffold materials (Fig. 2a). The thicknesses of the neocartilages were comparable to the native cartilage from femoral condyle (Fig. 2b). The tissues did not increase much in height during the cell culture period (Fig. 2b). After 6 weeks of cell culture, all the samples felt to be more resilient when cutting them for further analyses, compared to the earlier time-points. The pinkish color of the neotissues in the Fig. 2a is caused by phenol red used in the cell culture medium.

### Histological analysis

**Fig. 3:**
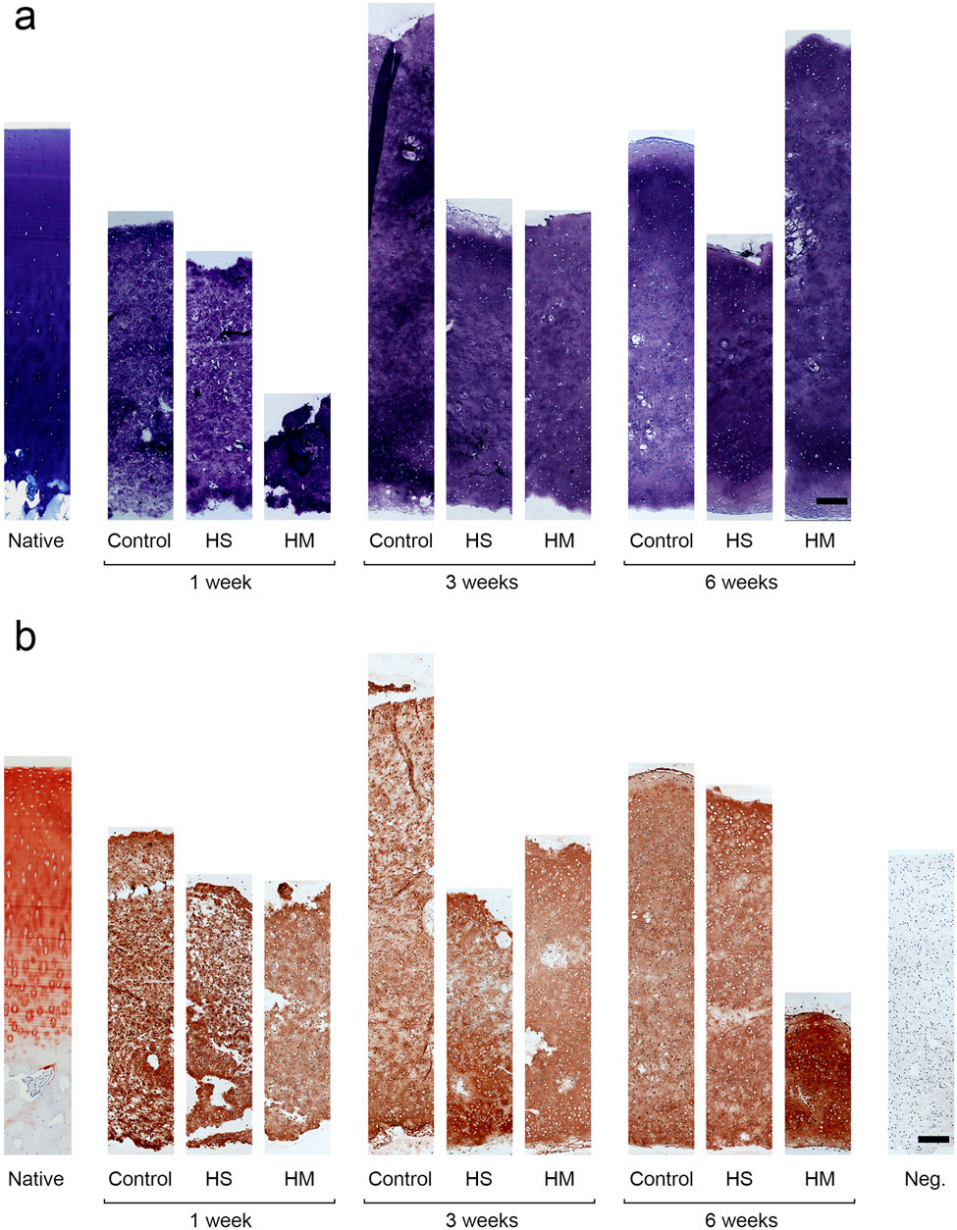
Microscopical appearance of the neocartilage tissues formed in the HyStem™ or the HydroMatrix™ scaffolds, or the scaffold-free (Control) cultures after 1, 3 or 6 weeks of culture. The proteoglycans **a** were stained with Toluidine Blue and type II collagens **b** were stained with an anti-type II collagen antibody (scale bar = 200 µm). *Native* native bovine articular cartilage *Neg*. negative control

The histological stainings were used to study the tissue structure of the neocartilages. Toluidine Blue staining showed no obvious differences in the intensity of the stainings between the neocartilages generated in the scaffolds and in the scaffold-free cell cultures at any of the three time points (Fig. 3a). The intensities of the PG staining were rather intense already after 1 week-cultivation, while it slightly increased following the cell culture period in all of the groups (Fig. 3a). The staining distributed quite evenly and intensively throughout the histological sections in all neocartilage tissues, although occasionally some empty, unstained areas could be observed (Fig. 3a). The staining intensities of the constructs were comparable to the native cartilage (Fig. 3a).

The type II collagen immunostainings showed that there were no clear differences in the staining intensities of the between the neocartilages generated in the scaffold-supported and the scaffold-free cultures, although the staining of the neocartilages generated in the HydroMatrix™ at 6 week time point appeared slightly stronger (Fig. 3b). The intensities of the type II collagen stainings were also similar to the native cartilage (Fig. 3b).

### Proteoglycan contents and subpopulations

**Fig. 4:**
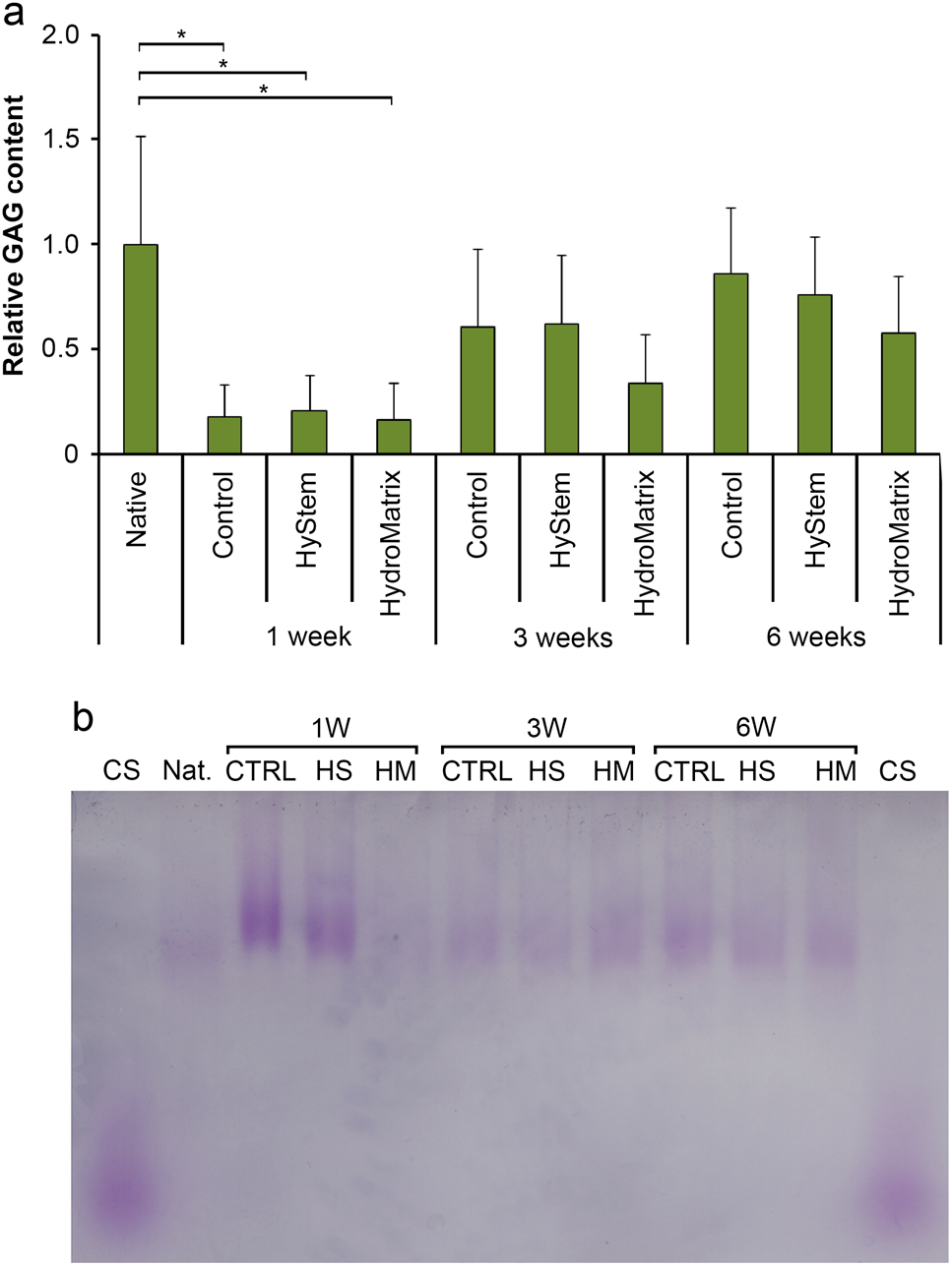
The relative glycosaminoglycan (GAG) contents (mean ± 95% confidence intervals, *n* = 4) and the PG structures in the neocartilage tissues generated in the HyStem™ or the HydroMatrix™ scaffolds, or the scaffold-free (Control) after 1, 3 or 6 weeks of culture. Dimethylmethylene blue assay was used to quantify the GAG contents and the results were normalized to the wet weight of the tissue. The quantities were related to native bovine articular cartilage’s GAG content **a** A representative figure of agarose gel used for electrophoretic separation of the PGs. The Toluidine Blue-stained agarose gel revealed the PG subpopulations of the neocartilages generated in the HyStem™ (HS) or the HydroMatrix™ (HM) scaffolds, or the scaffold-free (CTRL) cultures after 1, 3 or 6 weeks of culture **b** *CS* chondroitin sulfate, *Native* native bovine articular cartilage. Statistically significant differences (*P* < 0.05) are marked with *asterisks*

The contents of the GAGs increased in all neocartilages following the cell culture time. However, there were no statistically significant differences in the GAG contents of the neocartilages generated in the scaffold-supported or scaffold-free cell cultures at any time point except between the native and the samples cultured for 1 week [*p*-values: ctrl 0.037, HyStem™ 0.050 and HydroMatrix™ 0.031 (Fig. 4a)]. The GAG contents of the tissues after 6-week-cultivations were comparable to the content of native cartilage (Fig. 4a).

The agarose gel electrophoresis was used to analyze the size distribution of the PG subpopulations. In the Toluidine Blue-stained agarose gels, only slowly migrating bands, consisting of the large PGs corresponding to aggrecan, were observed in all samples (Fig. 4b). No consistent differences in the electrophoretic mobilities could be noticed, and no faster mobility bands, representing smaller PGs, were visible in any sample (Fig. 4b).

### Expressions of cartilage-specific genes and procollagen α_2_(I)

**Fig. 5:**
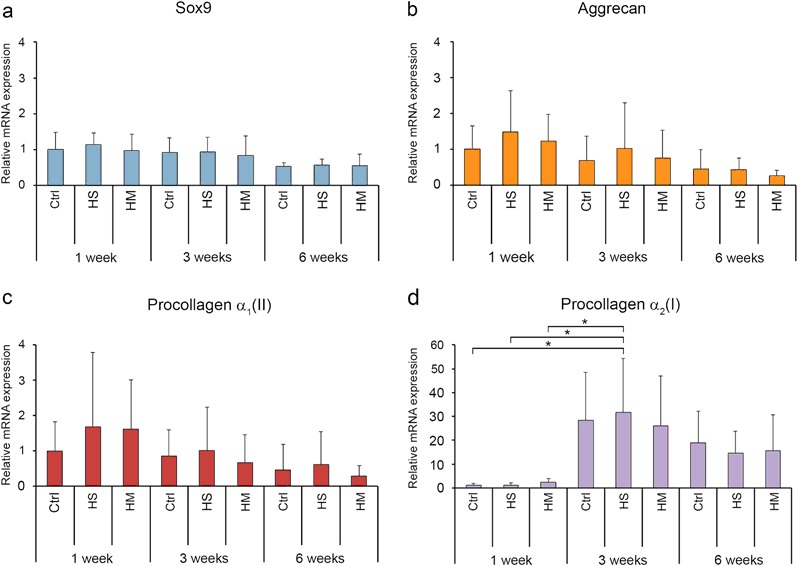
The mRNA expression levels (mean ± 95% confidence intervals, *n* = 7) of Sox9 **a** aggrecan **b** procollagen α_1_(II) **c** and procollagen α_2_(I) **d** in the neocartilages generated in the scaffold-free cultures (Control), or the HyStem™ or the HydroMatrix™ scaffold cultures after 1, 3 or 6 weeks of culture. The gene expressions were normalized to human Ribosomal Protein Large P0. Statistically significant differences (*P* < 0.05) are marked with *asterisks*

The quantitative RT-PCR was used to investigate the cartilage-specific genes aggrecan, procollagen α_1_(II), and Sox9, and also procollagen α_2_(I), mRNA expression levels in all samples (Fig. 5). There were no statistically significant differences in the cartilage-specific gene expressions upon the duration of the culture period (Fig. 5a–c), although the levels tended to be slightly higher at the beginning of the culture. Also, no differences were found between the different groups (Fig. 5a–c). However, the present study shows that the mRNA expression level of procollagen α_2_(I) remarkably increased after 1-week culture (Fig. 5d), showing the highest expression levels in constructs cultured for 3-weeks (Fig. 5d). There were statistically significant differences between the 3 week HyStem™ and the 1 week samples (*p*-values: control 0.021, HyStem™ 0.021 and HydroMatrix™ 0.033).

## Discussion

Scaffold-free tissue engineering utilizes culture methods, which do not use exogenous materials to support the manufacture of three-dimensional neotissues. Thus, this culture method can closely mimic the embryonic development, where a high cell density in the developing anlagen precedes matrix production [19], and to overcome certain limitations associated with the use of scaffolds [11, 20]. For instance, there are examples that neocartilages generated in the scaffold-free cell cultures can produce a better cartilage extracellular matrix, even closely similar to the native articular cartilage [12, 21–26]. However, the scaffold materials can be expected to shorten the time required for the formation of cartilage tissue with appropriate biological properties.

The present study was aimed to test whether the scaffolds made of the HyStem™ or the HydroMatrix™ would be beneficial over the scaffold-free cultures for the neocartilage formation in the agarose gel well-supported bovine primary chondrocyte cultures. Agarose wells were used in this study, since the plastic insert chondrocyte culture system we used in previous studies [6, 8] had certain limitations in generating an optimal cartilage-like tissue.

The HyStem™ biomaterial is a thiol-modified hyaluronan, which is the simplest GAG and an important component for extracellular matrix assembly of the cartilage [27]. Hyaluronan scaffolds have been used in cartilage tissue engineering to enhance the attachment, proliferation and differentiation of the chondrocytes [28, 29], and the assembly of the cartilage extracellular matrix [30]. Therefore, we hypothesized that the HyStem™ scaffold would be beneficial for the neocartilage formation in the agarose gel support chondrocyte culture system. The Hydromatrix™ was used in the present study since it has been shown to provide good three-dimensional matrix for the chondrocyte proliferation [31].

However, our finding was that neither of the scaffold materials was really superior to the scaffold-free culture. The contents of the PGs increased in all the neocartilage tissues generated either in the scaffold-supported or the scaffold-free cultures over time. The mRNA expression level of all the cartilage-specific genes [aggrecan, procollagen α_1_(II) and Sox9] gradually dropped during the 6-week culture period similarly in all the experimental groups. The HyStem™ or the HydroMatrix™ scaffolds did not show any benefit on the extracellular matrix production of the neocartilages. On the other hand, the increased mRNA expression of procollagen α_2_(I) occurred after 1-week cell culture.

The histological sections showed that the intensity of PG staining increased along the cell culture period, even though the cartilage specific-genes mRNA expression decreased. This apparently means that the expression of the hyaline cartilage-specific genes declines when the cartilaginous tissues have accumulated an adequate amount of extracellular matrix. The relatively high expression of procollagen α_2_(I) could raise a risk that the generated tissue would be fibrocartilage in nature. However, the histological sections did not show typical fibrocartilage patterns.

Many different approaches have been used for the scaffold-free cartilage tissue engineering, such as cell sheet engineering [20, 32], aggregate tissue engineering [21, 33], self-assembling process [12], and insert culture [6, 8, 23]. Of these approaches, the self-organization and the self-assembly have been the two most used categories [12]. Sufficient nutritional agents, such as glucose and growth factors, are also critical for the optimal neocartilage generation in the cell culture systems. Our previous study using the insert cell culture system showed that the PG staining was weaker in the middle part of the neocartilage tissue than that at the surface or in the deep zone close to the insert filter, which allows free diffusion of nutrients [6].

We assumed that we can improve the efficiency of nutrition transfer using the agarose gel-supported well system. Agarose system is also easy to handle, has a low cost and a minimal biological effect on the cells and regenerative tissues [34, 35]. The other advantage of using the agarose gels for the cell culture is that the nutrition or oxygen can freely diffuse through the agarose matrix. As shown by our present results, fine cartilage tissues closely comparable to native articular cartilage could be generated in the scaffold-free agarose gel-supported chondrocyte cultures. However, acellular areas could also be observed. Further, the agarose support could stably hold the assembly of the tissue-engineered constructs. Previously, it has been reported that 3-dimensional tissue-engineered cartilage generated in agarose mold has been used for clinical application [35].

We conclude that the scaffold-free agarose gel-support chondrocyte culture system generated a cartilage-like tissue, which is comparable to native cartilage. The agarose gel wells proved to be beneficial for the neocartilage formation. The tissues self-assembled in the gel wells were regularly shaped, and had smooth surface with hyaline cartilage–like appearance. However, no obvious benefits could be visible in the extracellular matrix production of the generated cartilage tissues in the HyStem™ or the HydroMatrix™ scaffold cultures. Since the decreased cartilage specific-genes and increased procollagen α_2_(I) mRNA expression levels seen in the generated neocartilage after 1-week cell culture, further investigations on testing some exogenous factors supplemented in the cell culture medium during the later culture time point are needed.
